# Personalized Diabetes Therapy Part 2—Individual Diabetes Treatment (Standard of Care Plus, SOC+)

**DOI:** 10.3390/jpm16040229

**Published:** 2026-04-20

**Authors:** Julia Jantz, Andreas Pfützner

**Affiliations:** 1 Pfützner Science & Health Institute, D-55128 Mainz, Germany; julia.jantz@pfuetzner-mainz.com; 2 Institute for Digital Technologies in Medicine & Dentistry, Institut Supérieur der Formation Continue, L-5393 Schuttrange, Luxembourg; 3 Department of Biotechnology & Bioinformatics, Technical University Bingen, D-55411 Bingen, Germany

**Keywords:** type 2 diabetes mellitus, functional biomarker, phenotyping, personalized diabetes treatment

## Abstract

Conventional diabetes therapy primarily targets HbA1c using a standardized, stepwise approach, often neglecting individual clinical and diagnostic phenotypes. In this second part of our discussion, we present an alternative strategy. After phenotyping the patient, we initiate a targeted pharmacological combination therapy tailored to the individual’s underlying pathophysiology, alongside lifestyle modifications. Sulfonylureas are completely avoided in this approach. Instead, medications are selected based on their alignment with the patient’s phenotype and absence of contraindications. Early insulin therapy, for example, is particularly effective in patients with β-cell-dysfunction-driven diabetes, whereas GLP-1-supported weight reduction and glitazone therapy are more suitable for insulin-resistance-driven diabetes. For monitoring and determining when temporary therapy intensification may be necessary, we rely on a combination of functional biomarkers (intact proinsulin, adiponectin, hsCRP, and leptin) and conventional clinical parameters (HbA1c, BMI, lipids, blood pressure). Using this personalized strategy, we have consistently achieved long-term glycemic control—often maintaining normal HbA1c levels for up to 15 years in our patients so far.

## 1. Introduction

Type 2 diabetes is a complex, multifactorial disease characterized by interrelated disorders such as β-cell dysfunction, insulin resistance, hormonal hyperactivity of visceral adipose tissue, and chronic systemic inflammation. Standard diabetes therapy, as currently practiced, focuses almost exclusively on glycemic control (HbA1c) and prevention of late-stage diabetic complications. It typically begins with lifestyle interventions, followed by metformin monotherapy, and gradually escalates to include multiple agents, culminating in insulin therapy only when other treatments fail [1,2,3].

However, this standardized treatment model does not sufficiently address the underlying pathophysiology of the disease. As a result, highly effective interventions—such as early insulin therapy—are seldom employed, or not used at all. Consequently, type 2 diabetes becomes a chronically progressive disease which, despite years of “HbA1c management,” often leads to premature death from macrovascular or microvascular complications. On average, it takes 10–14 years post-diagnosis before insulin is introduced, and the life expectancy of type 2 diabetes patients remains shortened [4,5,6,7].

In our practice, we have modified the standard care model by implementing a therapy approach based on prior phenotyping using functional biomarkers [8,9]. We prioritize pharmaceutical agents and combinations that specifically target the predominant underlying disorder—an approach we refer to as “Standard of Care Plus” (SOC+). While remaining aligned with clinical guidelines where possible, we do not hesitate to use early-stage combinations or drugs that are best suited to the patient’s individualized pathophysiological profile.

## 2. How Current Medications Affect the Pathophysiology of Type 2 Diabetes

All approved medications for diabetes mellitus reduce blood glucose levels (see Table 1), and lifestyle modifications can improve overall pathophysiological states when implemented effectively. In a purely glucose-centric treatment model, the conventional approach—beginning with metformin and adding sulfonylureas as needed—may indeed succeed in controlling blood glucose for several years. However, as outlined in Part 1 of our article [9,10], this strategy fails to address the deeper pathophysiological mechanisms, allowing macrovascular complications to develop insidiously.

It is now widely accepted that sulfonylureas, in particular, may accelerate disease progression and potentially cause additional harm. This concern will be explored further in the dedicated subsection on sulfonylureas. The following sections briefly evaluate each available class of antidiabetic drugs based on their impact on the fundamental pathophysiological components of type 2 diabetes.

### 2.1. Metformin

Metformin remains the universally accepted first-line pharmacological agent in national and international guidelines [1,3]. It primarily works by inhibiting hepatic gluconeogenesis, particularly reducing glucose production during fasting states [11,12,13]. Although various pleiotropic effects—including cardiovascular benefits—have been proposed, robust clinical evidence supporting these claims is limited.

A frequently cited subgroup analysis from the UKPDS trial involving 342 metformin-treated patients did demonstrate a significant reduction in cardiovascular mortality [14]. However, these participants were overweight at baseline and experienced weight loss during the trial—despite metformin’s confirmed inhibitory effect on lipolysis in vitro [15]. Most additional “cardioprotective” data for metformin are derived from comparisons with sulfonylureas, which themselves increase cardiovascular risk [16,17,18].

Only very few direct monotherapy comparisons exist between metformin and newer antidiabetic agents. One of them is the prospective ADOPT study, which compared sulfonylureas, metformin, and rosiglitazone in terms of glycemic durability. While metformin outperformed sulfonylureas, rosiglitazone proved significantly superior to metformin [19,20].

We therefore regard metformin primarily as a glucose-lowering agent with neutral or marginal effects on the underlying pathophysiology of diabetes. It should not be classified as a systemic insulin sensitizer, as often reported in the literature [21]. Notably, metformin does not influence vascular insulin receptors.

#### Side Effects and Safety Considerations for Metformin

Gastrointestinal side effects affect nearly half of all metformin-treated patients, particularly during treatment initiation or dose escalation [11,12]. Given the availability of better-tolerated alternatives (e.g., SGLT-2 inhibitors or DPP-IV inhibitors), we reserve metformin for situations where other options are contraindicated or when significantly elevated blood glucose levels necessitate its use.

### 2.2. Sulfonylureas and Glinides

We consider sulfonylureas (SUs) and glinides to be obsolete in modern diabetes management. Their mechanism—non-physiological stimulation of β-cells via sodium channel modulation—induces premature exhaustion of insulin secretory capacity and promotes negative disease progression.

Numerous high-quality studies confirm that sulfonylureas increase cardiovascular mortality [22,23]. A particularly illustrative meta-analysis of Canadian insurance data found that patient adherence to sulfonylurea therapy was paradoxically associated with increased mortality (hazard ratio: 1.3), while adherence to metformin showed no negative but also no positive effect [24].

Due to their adverse effects—and, to a lesser extent, those of glinides—we exclude both classes from our personalized treatment protocols.

### 2.3. DPP-IV Inhibitors

Dipeptidyl peptidase-4 (DPP-IV) inhibitors exert their therapeutic effect by inhibiting the degradation of incretin hormones—primarily glucagon-like peptide-1 (GLP-1) and gastric inhibitory polypeptide (GIP) [25]. These hormones are secreted postprandially and play a key role in modulating appetite and glycemic control [26].

By inhibiting DPP-IV, the breakdown of GLP-1 and GIP is slowed, leading to prolonged hormone activity. This results in increased glucose-dependent insulin secretion, reduced glucagon release [26,27], delayed gastric emptying [28], and decreased postprandial glucose excursions [26]. GLP-1 also contributes to satiety via hypothalamic signaling, and DPP-IV inhibitors may support weight regulation, albeit to a lesser degree than GLP-1 receptor agonists [29].

In our practice, DPP-IV inhibitors are primarily used to support GLP-1-based therapies, particularly in patients with an insulin-resistance-dominant phenotype and concurrent overweight or obesity. Their favorable tolerability profile also makes them an attractive option for long-term therapy.

#### Side Effects and Safety Considerations for DPP-IV Inhibitors

DPP-IV inhibitors appear to be generally well tolerated; the adverse events reported in clinical trials are predominantly mild and nonspecific, including headache, nasopharyngitis, and gastrointestinal complaints [25,26]. This favorable tolerability profile supports their use as adjunctive long-term therapy in carefully selected patients.

### 2.4. Glitazones

Glitazones, or thiazolidinediones (TZDs), are peroxisome proliferator-activated receptor gamma (PPARγ) agonists. They enhance insulin signaling in peripheral tissues and the vasculature, thereby improving insulin sensitivity, especially in muscle and adipose tissue [29]. This results in increased glucose uptake and reduced blood glucose levels [30,31]. Glitazones also suppress hepatic gluconeogenesis [32,33].

Unlike rosiglitazone, pioglitazone offers additional metabolic benefits as it redistributes body fat by promoting subcutaneous fat storage and reducing visceral fat deposition in the liver and muscles [34,35,36]. It also reduces triglyceride levels [31,37], lowers small dense LDL particles [38,39], and raises HDL cholesterol [35,37], and it is also effective in treating non-alcoholic steatohepatitis (NASH) [40].

For us, the most important therapeutic attribute of pioglitazone is its anti-inflammatory effect, which mitigates chronic systemic inflammation. In one of our studies, pioglitazone reduced the activation of peripheral monocytes/macrophages—measured by the expression of pro-inflammatory cytokines—by approximately one-third within just three days [41]. This has been associated with improved cardiovascular risk profiles [42,43,44,45], and reduced intima–media thickness, a marker of atherosclerosis [46,47,48]. Thus, pioglitazone is our preferred TZD and a cornerstone of therapy for both the insulin resistance and chronic systemic inflammation (CSI) phenotypes.

#### Side Effects and Safety Considerations for Glitazones

The most commonly reported side effect of pioglitazone is fluid retention, which may lead to short-term weight gain, exacerbation of pre-existing heart failure, and peripheral edema. Additionally, an increased risk of bone fractures—particularly in postmenopausal women with osteoporosis—has been observed with long-term use [49]. These effects are attributed to improved insulin action and are also seen with the initiation of insulin therapy [50,51,52,53]. Cellular hydration following enhanced glucose uptake can also influence mood positively, especially in patients with depressive symptoms [54,55,56,57,58].

Although reimbursement for pioglitazone in Germany is limited due to historical concerns about bladder cancer risk [59,60], recent re-analyses have revealed that the original studies had significant methodological flaws [61,62]. Updated evidence has shown no increased risk of bladder cancer from pioglitazone use [63,64].

### 2.5. GLP-1 Agonists

Glucagon-like peptide-1 (GLP-1) receptor agonists mimic the effects of endogenous GLP-1, a key incretin hormone that regulates metabolism and appetite [65,66]. These agents enhance glucose-dependent insulin secretion [67,68,69], reduce cardiovascular risk [70,71], suppress glucagon release [72], delay gastric emptying [65,66], and promote satiety via hypothalamic receptors [73,74]. In contrast to sulfonylureas, GLP-1 agonists carry a low risk of hypoglycemia due to their glucose-dependent mechanism of action. Cardiovascular outcome trials (e.g., the LEADER study with liraglutide) have demonstrated significant cardiovascular benefits for certain GLP-1 agonists [70,71]. These medications are typically administered subcutaneously, with varying durations of action (daily vs. weekly formulations). In our practice, we favor GLP-1 agonists in patients requiring weight loss, especially those with insulin resistance or chronic systemic inflammation (CSI) phenotypes.

#### 2.5.1. Short-Acting vs. Long-Acting GLP-1 Agonists

We prefer short-acting GLP-1 agonists (e.g., exenatide, lixisenatide, liraglutide) over long-acting formulations (e.g., once-weekly exenatide, semaglutide). Our rationale is grounded in physiology: endogenous GLP-1 levels rise briefly after meals and are rapidly degraded [74,75]. Continuous elevation of GLP-1 via long-acting agents may lead to hypothalamic desensitization, diminishing the satiety effect over time. Upon discontinuation, patients may experience rebound hyperphagia and rapid weight regain. In contrast, short-acting agents more closely mimic natural physiology, reducing the risk of habituation. In the DURATION-6 study, liraglutide (short-acting) demonstrated superior weight reduction compared to once-weekly exenatide (–3.6 kg vs. –2.7 kg over 6 months) [76,77]. We are currently evaluating emerging dual incretin therapies (e.g., tirzepatide, a GIP/GLP-1 receptor agonist) to determine their role in our personalized treatment approach.

##### Side Effects and Safety Considerations for GLP-1 Agonists

Nausea, vomiting, and diarrhea are the most common adverse effects of GLP-1 receptor agonists, particularly during initiation and dose escalation, but they are usually transient and dose-dependent [65,66,72].

### 2.6. SGLT-2 Inhibitors

Sodium-glucose cotransporter-2 (SGLT-2) inhibitors lower blood glucose by inhibiting renal glucose reabsorption, resulting in increased urinary glucose excretion [78,79]. Under normal conditions, glucose is reabsorbed by the kidneys up to a plasma threshold of approximately 180 mg/dL. When this threshold is surpassed, glucose becomes cytotoxic, and its renal excretion is a protective mechanism—a concept known since antiquity [80].

By lowering the renal threshold, SGLT-2 inhibitors facilitate glucose excretion even at normoglycemic levels, leading to a loss of approximately 300–400 kcal/day. This contributes to weight reduction [81], reduced visceral adipose tissue hormonal activity, lower circulating angiotensin II levels [82], and improved blood pressure due to osmotic diuresis and sodium loss [83,84].

#### 2.6.1. Cardiorenal Benefits

Recent studies have confirmed that SGLT-2 inhibitors reduce major adverse cardiovascular events, hospitalization for heart failure, and the progression of chronic kidney disease [85,86]. These benefits have led to the inclusion of SGLT-2 inhibitors in heart failure treatment guidelines, extending their use beyond glycemic control [87].

In our personalized treatment protocols, we regularly incorporate SGLT-2 inhibitors as an adjunctive therapy—especially for supporting weight loss, lowering blood glucose, and enhancing the effects of GLP-1 agonists or pioglitazone.

##### Side Effects and Safety Considerations for SGLT-2 Inhibitors

The most common adverse events include genital and urinary tract infections due to increased glucose in the urine, while a rare but serious complication is euglycemic diabetic ketoacidosis, especially under conditions of dehydration, low carbohydrate intake, or insulin deficiency [85,88]. Appropriate patient selection and education are therefore crucial to minimizing risks.

### 2.7. Insulin—An Ambivalent Hormone

In most current diabetes guidelines, insulin is considered a last-resort therapy—introduced only after failure of lifestyle measures, metformin, and multiple other agents [1]. However, this conservative approach overlooks the considerable therapeutic potential of insulin, particularly when applied early and temporarily in disease progression [89].

Numerous studies have shown that short-term intensive insulin therapy—lasting 2–3 weeks—can induce prolonged glycemic remission, allowing some patients to maintain normoglycemia without continued pharmacologic intervention [90]. This strategy is especially widespread in Asia and has proven particularly effective in patients with a β-cell dysfunction (BCD) phenotype. For example, Li et al. demonstrated that temporary intensive insulin therapy can restore first-phase insulin secretion and improve β-cell function [91].

In our approach, we often introduce low-dose basal insulin early to support β-cell recovery. This is typically combined with oral antidiabetic agents to preserve or even enhance endogenous insulin production. The ADA/EASD consensus also recognizes temporary insulin therapy as an option for patients with HbA1c ≥ 9%, and recommends it strongly for those with HbA1c ≥ 10–12% [92]. National guidelines, such as those from Israel, support similar recommendations [93].

Recent data from the U.S. show that 23% of newly diagnosed patients present with an HbA1c ≥ 9% at treatment initiation [94], underscoring the need for early, aggressive intervention in a significant patient subset.

#### 2.7.1. Vascular Effects of Insulin

The endothelial insulin receptor mediates vasoprotective effects through activation of endothelial nitric oxide synthase (eNOS), resulting in nitric oxide (NO) production. NO has antioxidant properties and protects blood vessels from postprandial oxidative stress.

However, in the setting of insulin resistance, abnormal insulin signaling (wrong dose, wrong kinetics) activates the MAP kinase pathway, promoting secretion of endothelin-1, a pro-inflammatory cytokine. This leads to atherogenic effects, especially if insulin is introduced too late in the disease course [89].

#### 2.7.2. Pulsatile vs. Continuous Insulin Secretion

Physiological insulin secretion occurs in low-frequency pulses (10/h). These pulses are lost early in both type 1 and type 2 diabetes [95,96]. This contributes to microvascular complications, which occur at similar rates in both types of diabetes. Restoring pulsatile insulin exposure has shown promising benefits by delaying renal function decline in type 1 diabetes [97,98,99] and improving kidney function in patients with type 2 diabetes [100]. Therefore, early and appropriately dosed insulin—preferably mimicking physiological pulsatility—may not only normalize glucose but also exert vascular protective effects.

## 3. From Phenotype to Treatment

In our personalized diabetes therapy model, we apply specific interventions that target the dominant underlying pathophysiological mechanisms of each patient. Whether the therapy involves a single agent or a drug combination, it is always paired with effective lifestyle modification, which we regard as essential to long-term success.

### 3.1. Lifestyle Recommendations

Based on our pathophysiological rationale, we recommend a low-carbohydrate, high-fiber, and high-protein diet. This dietary approach has been shown to reduce glucose spikes and lower blood sugar levels [101], improve insulin sensitivity [102], support weight loss [103], lower HbA1c and other metabolic risk markers [104], and reduce the need for antidiabetic and comorbidity-related medications [105].

We also encourage a comprehensive exercise regimen that includes aerobic training: ≥150 min per week of moderate-intensity activity for cardiovascular health and glucose control [106], resistance training: 2–3 sessions per week to enhance muscle mass and metabolic function [107], as well as flexibility and balance training (especially for older adults to reduce the risk of falls and improve mobility) [108].

### 3.2. Pharmacologic Strategy

**Figure 1:**
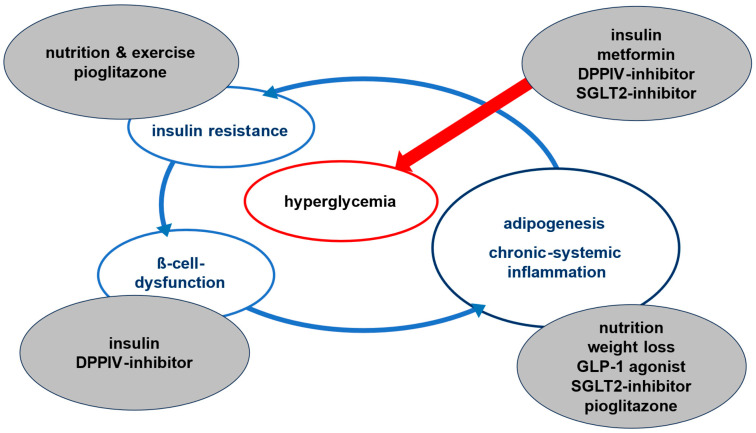
Phenotype-specific treatment framework derived from Table 1. The figure illustrates how the dominant pathophysiological driver (β-cell dysfunction, insulin resistance, or chronic systemic inflammation) guides selection of preferred first-choice and adjunctive therapies within the SOC+ concept.

We support early use of rational drug combinations, especially in cases where metformin intolerance is documented—a practice consistent with current diabetes guidelines [1,2,3]. Every approved diabetes medication lowers blood glucose to some extent, but their impact on underlying pathophysiology varies widely. To make our therapeutic reasoning more transparent, Figure 1 summarizes how we translate the dominant phenotype into phenotype-specific first-choice and adjunctive drug selections in daily practice.

### 3.3. General Considerations for Personalized Treatment Selection

Thus far, we have focused on our diagnostic phenotyping model and the mechanisms of action of various antidiabetic agents, as implemented in our local German practice environment. This includes access to reimbursed biomarker testing (e.g., intact proinsulin, adiponectin), which may not be readily available or economically viable in all healthcare systems. Nonetheless, several additional factors—independent of biomarker access—must be considered when selecting an optimized, personalized treatment strategy, including but not limited to drug contraindications (e.g., renal function, hepatic disease, heart failure), potential side effects and patient tolerability, patient preferences (e.g., oral vs. injectable therapy), economic and reimbursement conditions in the local healthcare system, drug availability in specific countries, and cultural and genetic differences, which may affect drug response.

Our approach has been successfully applied across a broad range of ethnicities, including patients from China, India, Africa, and the Arab Peninsula, the United States (including African American and Latino populations), and various socio-economic backgrounds in Germany. It is worth noting that we briefly operated a satellite clinic in Cairo, Egypt (2016–2017), where our model also proved effective—albeit predominantly among patients with the means to travel or access private care. We recognize that in certain situations, access to the “theoretically optimal” medication may be limited. In such cases, we recommend using the next-best intervention, guided by Table 1 and Figure 1. Metformin may be used more frequently when cost constraints apply, as it remains inexpensive and offers modest benefits on hepatic insulin resistance and inflammation. However, sulfonylureas should be avoided altogether. Their use cannot be justified even in low-cost settings due to the significant increase in cardiovascular mortality and overall risk of early death, even among adherent patients [21,22,23].

Contemporary work in diabetes precision medicine has underscored that “type 2 diabetes” comprises biologically heterogeneous subgroups with different trajectories and complication risks. Data-driven clustering approaches using readily available clinical variables have repeatedly identified reproducible subphenotypes such as severe insulin-deficient and severe insulin-resistant patterns, alongside milder obesity-related and age-related forms [109,110,111].

Our SOC+ approach is conceptually aligned with these frameworks in that it translates heterogeneity into a mechanism-oriented bedside workflow: after excluding relevant alternative etiologies when indicated, we assign a dominant pathophysiological driver pattern (β-cell dysfunction, insulin resistance/visceral adipose dysfunction, or chronic systemic inflammation) to guide early combination therapy choices within guideline-compatible care.

At the same time, we recognize important implementation constraints. Some phenotyping biomarkers may not yet be routinely available in all countries, and access to certain preferred agents may be limited by reimbursement rules, regulatory restrictions, or local formularies. In these situations, our pragmatic recommendation is to select the next-best available intervention that best matches the dominant phenotype while respecting contraindications, tolerability, patient preferences, and economic realities.

## 4. Case Studies from Our Practice

To illustrate the procedures that is followed in our concept, we are providing several single case studies (the patients have approved the anonymous presentation of their data).

### 4.1. Case 1: Patient AP

- Sex/Age: Male, 69 years.
- Initial Visit: 15 July 2015.
- Diabetes Duration: 5 years.
- Anthropometrics: Height 176 cm, Weight 103.2 kg, BMI 33.2 kg/m^2^.
- Initial HbA1c: 7.5% (target <6.5%).
- Current Treatment: Dapagliflozin monotherapy (10 mg), discontinued metformin due to intolerance.

Phenotyping Results at Baseline:

- Intact Proinsulin: 10.5 pmol/L (normal <7 pmol/L).
- Adiponectin: 4.1 mg/L (normal in men >6 mg/L).
- hsCRP: 2.71 mg/L (normal <1 mg/L).

Interpretation: The patient exhibited predominantly insulin-resistance-driven diabetes with stage III β-cell dysfunction, marked insulin resistance, and moderately elevated cardiovascular risk. Because metformin had already been discontinued for intolerance and the biomarker profile pointed primarily toward insulin resistance rather than isolated hyperglycemia, we selected pioglitazone as the key phenotype-directed intervention and used an SGLT-2 inhibitor initially to support glycemic control and weight-related metabolic stress.

Therapeutic Intervention: Pioglitazone 45 mg was added to dapagliflozin. After two urinary tract infections associated with SGLT-2 inhibitor exposure (one in 2015 with dapagliflozin and another in 2019 with empagliflozin), treatment was individualized further and switched to saxagliptin 5 mg. This change illustrates our practical treatment algorithm: maintain the phenotype-targeted core therapy when effective, but adapt the accompanying drug class according to tolerability and safety.

**Figure 2:**
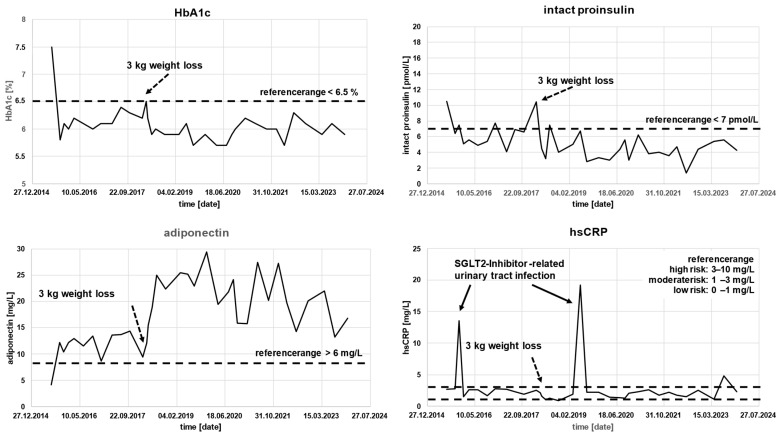
Patient AP, 69 years old, course of HbA1c and phenotyping parameters in the years 2015–2024 under the optimized SOC+ combination therapy (pioglitazone 45 mg/saxagliptin 5 mg).

Outcome: All biomarker parameters normalized and remained stable (see Figure 2), HbA1c remained consistently within target range, body weight remained stable between 99–103 kg; current weight (April 2025): 98.5 kg and there was no evidence of diabetes progression over 10 years of follow-up

### 4.2. Case 2: Patient VLT

- Sex/Age: Female, 62 years.
- Initial Visit: August 2012 (new diagnosis).
- Anthropometrics: Height 175 cm, Weight 94 kg, BMI 30.7 kg/m^2^.
- Initial HbA1c: 10.2%.
- Prior Therapy:
  1. Metformin 500 mg (discontinued due to GI side effects).
  2. Switched to Glimepiride 3 mg.

Phenotyping Results at Baseline:

- Intact Proinsulin: 14.5 pmol/L (normal <7 pmol/L).
- Adiponectin: 2.1 mg/L (normal in women >8 mg/L).
- hsCRP: 6.4 mg/L (high-risk range).

Interpretation: This patient presented with a combined CSI/ßCD-driven phenotype characterized by insulin resistance, chronic systemic inflammation with high cardiovascular risk, and qualitatively impaired β-cell secretion (stage III). Because the phenotype was mixed rather than purely hyperglycemic, we chose a combination intended to address several mechanisms simultaneously: incretin support for β-cell function and appetite regulation, together with pioglitazone for insulin resistance and inflammation.

Therapeutic Intervention: Initial combination therapy consisted of liraglutide 0.6 mg (increased to 1.2 mg after 2 weeks), pioglitazone 30 mg, and sitagliptin 50 mg. We selected this GLP-1 receptor agonist/DPP-IV inhibitor combination to provide prolonged incretin activity throughout the day, while pioglitazone was added to address the pronounced insulin resistance and inflammatory phenotype.

**Figure 3:**
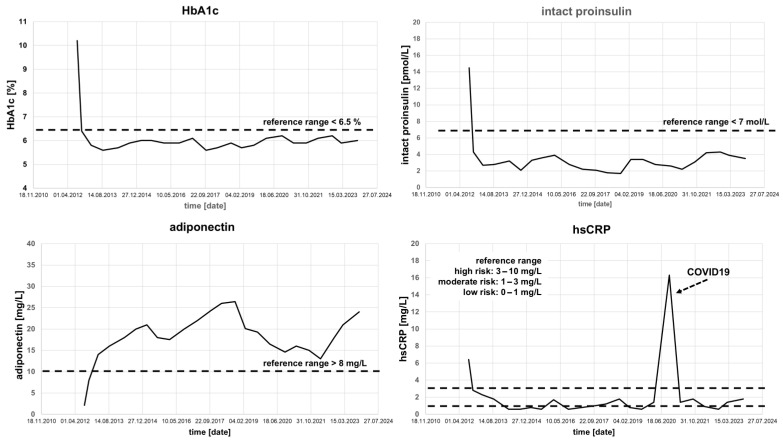
Patient VLT, 62 years old, course of HbA1c and phenotyping parameters in the years 2012–2023 under the optimized SOC+ combination therapy (liraglutide 1.2 mg, pioglitazone 30 mg, sitagliptin 50 mg).

Outcome: HbA1c normalized and remained stable for over 11 years (see Figure 3), and body weight decreased from 94 kg to 73 kg (BMI from 30.7 to 23.5 kg/m^2^). The patient suffered from a mild SARS-CoV-2 infection in 2020; otherwise, no major health events occurred. There was no evidence of diabetes progression or deterioration of biomarker profiles as of April 2025.

**Figure 4:**
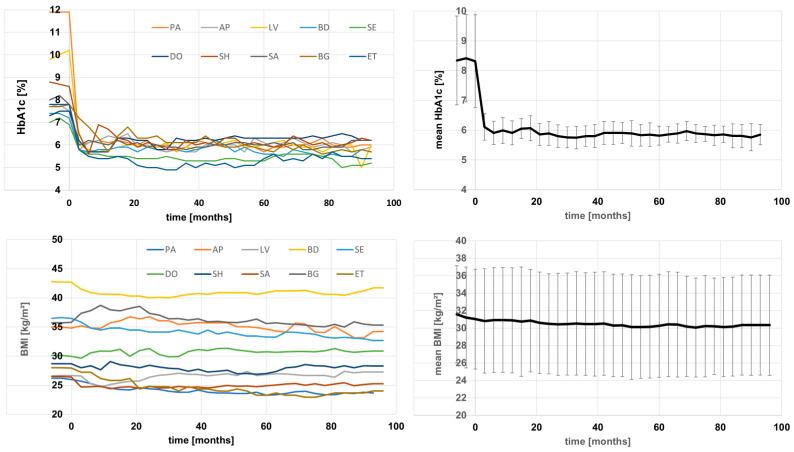
Individual patient trajectories and mean course for the first 10 patients treated with the personalized treatment concept for 96 months (5 female/5 male; age: 66.5 ± 8.9 years; baseline BMI: 31.0 ± 5.7 kg/m^2^; baseline HbA1c: 8.3 ± 1.5%; after 96 months BMI: 30.3 ± 5.8 kg/m^2^, *p* vs. baseline < 0.05; HbA1c: 5.9 ± 0.3%, *p* vs. baseline < 0.001).

These two case studies illustrate how the SOC+ model is operationalized in practice: phenotype assignment determines the initial therapeutic direction, while longitudinal biomarker and clinical follow-up determine whether treatment should be intensified, simplified, or modified for tolerability. Since 2006, we have treated more than 200 patients with this approach. After initial therapy optimization and adjustment for tolerability, most patients have remained stable over multi-year treatment periods unless disrupted by major external events such as severe illness, substantial weight gain, or acute stress. To complement the two detailed index cases, Figure 4 summarizes the first 10 patients treated for more than 8 years with this concept.

Temporary additions of insulin are occasionally recommended in the context of acute increases in insulin resistance (e.g., postoperative states, major illness), with the aim of rapidly reversing stress-related metabolic deterioration.

## 5. Conclusions and Considerations

In our clinical practice, we aim to prevent progression of type 2 diabetes and its long-term complications through systematic phenotyping with functional biomarkers and through targeted, individualized treatment strategies. The cases and long-term follow-up data presented here suggest that this SOC+ approach can support durable glycemic control and metabolic stabilization when the dominant pathophysiological driver is identified early and therapy is aligned accordingly.

A central practical message of our work is that personalized diabetes therapy should not be limited to selection of a single glucose-lowering drug. Rather, it should combine phenotype-guided drug choice, structured lifestyle intervention, longitudinal reassessment of biomarkers and clinical parameters, and timely adaptation for efficacy, tolerability, contraindications, and local access conditions. If residual β-cell function remains, individualized temporary intensification or de-escalation strategies may even allow partial remission in selected patients [112].

Our intention in presenting this concept is not to claim definitive proof over all other care models, but to offer a pragmatic, pathophysiology-oriented framework that can be tested, refined, and prospectively evaluated in broader clinical settings. We hope that this debate article will encourage clinicians to further explore personalized, mechanism-based alternatives to purely glucocentric diabetes care.

## Figures and Tables

**Table 1:** Influence of anti-diabetic interventions on the pathophysiological underlying diabetic disorders (BG: blood glucose, ßCD: ß-cell dysfunction, IR: insulin resistance, CSI: chronic systemic inflammation).

Intervention	BG	ßCD	IR	CSI
Lifestyle (Nutrition & Exercise)	++	++	+	+
Weight loss	++	++	++	++
Metformin	++	0	(+)	(+)
Sulfonylurea	+++	−	−	−
Glinide	+++	−	−	−
DPPIV-Inhibitor	++	++	0	(+)
Glitazone	+	(+)	+++	+++
SGLT2-Inhibitor	++	+	+	++
GLP-1 Agonist	++	++	+	+
Insulin (early)	+++	+++	0	(+)
Insulin (late)	+++	+	−	−

Note: Currently emerging agents (e.g., tirzepatide) are not yet included. (“(+)”, “+”, “++”, and “+++” indicate a weak positive, positive, very positive and strong positive impact; “0” = no impact, “−” = negative impact).

## Data Availability

No new data were created or analyzed in this study.

## References

[1] American Diabetes Association Professional Practice Committee (2024). 15. Management of Diabetes in Pregnancy: Standards of Care in Diabetes-2024. Diabetes Care.

[2] Bundesärztekammer (BÄK), Kassenärztliche Bundesvereinigung (KBV), Arbeitsgemeinschaft der Wissenschaftlichen Medizinischen Fachgesellschaften (AWMF) (2021). Nationale VersorgungsLeitlinie Typ-2-Diabetes—Teilpublikation der Langfassung, 2. Auflage. Version 1. https://www.ddg.info/fileadmin/user_upload/05_Behandlung/01_Leitlinien/Evidenzbasierte_Leitlinien/2021/diabetes-2aufl-vers1.pdf.

[3] Landgraf R., Aberle J., Birkenfeld A.L., Gallwitz B., Kellerer M., Klein H.H., Müller-Wieland D., Nauck M.A., Wiesner T., Siegel E. (2020). Therapie des Typ-2-Diabetes [Treatment of type 2 diabetes]. Diabetologie.

[4] Ringborg A., Lindgren P., Yin D.D., Martinell M., Stålhammar J. (2010). Time to insulin treatment and factors associated with insulin prescription in Swedish patients with type 2 diabetes. Diabetes Metab..

[5] Salvador D., Bano A., Wehrli F., Gonzalez-Jaramillo V., Laimer M., Hunziker L., Muka T. (2023). Impact of type 2 diabetes on life expectancy and role of kidney disease among inpatients with heart failure in Switzerland: An ambispective cohort study. Cardiovasc. Diabetol..

[6] Bernstein S., Gilson S., Zhu M., Nathan A.G., Cui M., Press V.G., Shah S., Zarei P., Laiteerapong N., Huang E.S. (2023). Diabetes Life Expectancy Prediction Model Inputs and Results From Patient Surveys Compared With Electronic Health Record Abstraction: Survey Study. JMIR Aging.

[7] (2023). Emerging Risk Factors Collaboration. Life expectancy associated with different ages at diagnosis of type 2 diabetes in high-income countries: 23 million person-years of observation. Lancet Diabetes Endocrinol..

[8] Pfützner A., Weber M.M., Forst T. (2008). A Biomarker Concept for Assessment of Insulin resistance, ß-Cell Function and Chronic Systemic Inflammation in Type 2 Diabetes mellitus. Clin. Lab..

[9] Pfützner A. (2023). “Glukosekosmetik” vs. Personalisierte Diabetestherapie Teil 1: Funktionelle Phänotypisierung als Grundlage einer individuellen Typ 2 Diabetes Therapie. Diabetes Stoffw. Herz.

[10] Pfützner A. (2024). “Glukosekosmetik” vs. personalisierte Diabetestherapie Teil 2: Personalisierte Therapie. Diabetes Stoffw. Herz.

[11] Bailey C.J., Turner R.C. (1996). Metformin. N. Engl. J. Med..

[12] Frayn K.N., Adnitt P.I., Turner P. (1971). The hypoglycaemic action of metformin. Postgrad. Med. J..

[13] Hundal R.S., Krssak M., Dufour S., Laurent D., Lebon V., Chandramouli V., Inzucchi S.E., Schumann W.C., Petersen K.F., Landau B.R. (2000). Mechanism by which metformin reduces glucose production in type 2 diabetes. Diabetes.

[14] UK Prospective Diabetes Study (UKPDS) Group (1998). Effect of intensive blood-glucose control with metformin on complications in overweight patients with type 2 diabetes (UKPDS34). Lancet.

[15] Zhang T., He J., Xu C., Zu L., Jiang H., Pu S., Guo X., Xu G. (2009). Mechanisms of metformin inhibiting lipolytic response to isoproterenol in primary rat adipocytes. J. Mol. Endocrinol..

[16] Han Y., Xie H., Liu Y., Gao P., Yang X., Shen Z. (2019). Effect of metformin on all-cause and cardiovascular mortality in patients with coronary artery diseases: A systematic review and an updated meta-analysis. Cardiovasc. Diabetol..

[17] Hermann L.S., Ranstam J., Vaaler S., Melander A. (1999). Effects of antihyperglycaemic therapies on proinsulin and relation between proinsulin and cardiovascular risk factors in type 2 diabetes. Diabetes Obes. Metab..

[18] Li T., Providencia R., Jiang W., Liu M., Yu L., Gu C., Chang A.C.Y., Ma H. (2022). Association of Metformin with the Mortality and Incidence of Cardiovascular Events in Patients with Pre-existing Cardiovascular Diseases. Drugs.

[19] Kahn S.E., Haffner S.M., Heise M.A., Herman W.H., Holman R.R., Jones N.P., Kravitz B.G., Lachin J.M., O’Neill M.C., Zinman B. (2006). Glycemic durability of rosiglitazone, metformin, or glyburide monotherapy. N. Engl. J. Med..

[20] Kahn S.E., Lachin J.M., Zinman B., Haffner S.M., Aftring R.P., Paul G., Kravitz B.G., Herman W.H., Viberti G., Holman R.R. (2011). Effects of rosiglitazone glyburide metformin on β-cell function insulin sensitivity in ADOPT. Diabetes.

[21] Bailey C.J. (2024). Metformin: Therapeutic profile in the treatment of type 2 diabetes. Diabetes Obes. Metab..

[22] Bain S., Druyts E., Balijepalli C., Baxter C.A., Currie C.J., Das R., Donnelly R., Khunti K., Langerman H., Leigh P. (2017). Cardiovascular events and all-cause mortality associated with sulphonylureas compared with other antihyperglycaemic drugs: A Bayesian meta-analysis of survival data. Diabetes Obes. Metab..

[23] Forst T., Hanefeld M., Jacob S., Moeser G., Schwenk G., Pfützner A., Haupt A. (2013). Association of sulphonylurea treatment with all-cause and cardiovascular mortality: A systematic review and meta-analysis of observational studies. Diabetes Vasc. Dis. Res..

[24] Simpson S.H., Majumdar S.R., Tsuyuki R.T., Eurich D.T., Johnson J.A. (2006). Dose-response relation between sulfonylurea drugs and mortality in type 2 diabetes mellitus: A population-based cohort study. CMAJ.

[25] Holst J.J., Deacon C.F. (1998). Inhibition of the activity of dipeptidyl-peptidase IV as a treatment for type 2 diabetes. Diabetes.

[26] Deacon C.F., Lebovitz H.E. (2020). Dipeptidyl peptidase 4 inhibitors in the treatment of type 2 diabetes mellitus. Nat. Rev. Endocrinol..

[27] Chai S., Zhang Y., Carr R.D., Deacon C.F., Zheng C., Rajpathak S., Chen L., Yu Y. (2023). Impact of dipeptidyl peptidase-4 inhibitors on glucose-dependent insulinotropic polypeptide in type 2 diabetes mellitus: A systematic review and meta-analysis. Front. Endocrinol..

[28] McIntosh C.H., Demuth H.U., Pospisilik J.A., Pederson R. (2005). Dipeptidyl peptidase IV inhibitors: How do they work as new antidiabetic agents?. Regul. Pept..

[29] Hansen H.H., Hansen G., Paulsen S., Vrang N., Mark M., Jelsing J., Klein T. (2014). The DPP-IV inhibitor linagliptin and GLP-1 induce synergistic effects on body weight loss and appetite suppression in the diet-induced obese rat. Eur. J. Pharmacol..

[30] Forman B.M., Chen J., Evans R.M. (1996). The peroxisome proliferator-activated receptors: Ligands and activators. Ann. N. Y. Acad. Sci..

[31] Giglio R.V., Papanas N., Rizvi A.A., Ciaccio M., Patti A.M., Ilias I., Pantea Stoian A., Sahebkar A., Janez A., Rizzo M. (2022). An Update on the Current and Emerging Use of Thiazolidinediones for Type 2 Diabetes. Medicina.

[32] Barnett A.H. (2002). Insulin-sensitizing agents—Thiazolidinediones (glitazones). Curr. Med. Res. Opin..

[33] Ravikumar B., Gerrard J., Dalla Man C., Firbank M.J., Lane A., English P.T., Cobelli C., Taylor R. (2008). Pioglitazone decreases fasting and postprandial endogenous glucose production in proportion to decrease in hepatic triglyceride content. Diabetes.

[34] Shannon C.E., Daniele G., Galindo C., Abdul-Ghani M.A., DeFronzo R.A., Norton L. (2017). Pioglitazone inhibits mitochondrial pyruvate metabolism and glucose production in hepatocytes. FEBS J..

[35] Kodama N., Tahara N., Tahara A., Honda A., Nitta Y., Mizoguchi M., Kaida H., Ishibashi M., Abe T., Ikeda H. (2013). Effects of pioglitazone on visceral fat metabolic activity in impaired glucose tolerance or type 2 diabetes mellitus. J. Clin. Endocrinol. Metab..

[36] Hurren K.M., Dunham-Snary K.J., Bradford A.P. (2021). Are thiazolidinediones a preferred drug treatment for type 2 diabetes mellitus and associated comorbidities? An update. Expert Opin. Drug Metab. Toxicol..

[37] Miyazaki Y., Mahankali A., Matsuda M., Mahankali S., Hardies J., Cusi K., Mandarino L.J., DeFronzo R.A. (2002). Effect of pioglitazone on abdominal fat distribution and insulin sensitivity in type 2 diabetic patients. J. Clin. Endocrinol. Metab..

[38] Forst T., Wilhelm B., Pfützner A., Fuchs W., Lehmann U., Schaper F., Weber M., Müller J., Konrad T., Hanefeld M. (2008). Investigation of the vascular and pleiotropic effects of atorvastatin and pioglitazone in a population at high cardiovascular risk. Diabetes Vasc. Dis. Res..

[39] Hanefeld M., Marx N., Pfützner A., Baurecht W., Lübben G., Karagiannis E., Stier U., Forst T. (2007). Anti-inflammatory effects of pioglitazone and/or simvastatin in high cardiovascular risk patients with elevated high sensitivity C-reactive protein: The PIOSTAT Study. J. Am. Coll. Cardiol..

[40] Leonhardt W., Pfützner A., Müller J., Pietzsch J., Forst T., Karagiannis E., Lübben G., Hanefeld M. (2008). Effects of pioglitazone and/or simvastatin on low density lipoprotein subfractions in non-diabetic patients with high cardiovascular risk: A sub-analysis from the PIOSTAT study. Atherosclerosis.

[41] Deng M., Wen Y., Yan J., Fan Y., Wang Z., Zhang R., Ren L., Ba Y., Wang H., Lu Q. (2023). Comparative effectiveness of multiple different treatment regimens for nonalcoholic fatty liver disease with type 2 diabetes mellitus: A systematic review and Bayesian network meta-analysis of randomised controlled trials. BMC Med..

[42] Pfützner A., Weise A., Pfützner-Riehn E., Lübben G., Morcos M., Karagiannis E., Weber M., Forst T. (2011). Downregulation of the proinflammatory state of circulating mononuclear cells by short-term treatment with pioglitazone in patients with type 2 diabetes mellitus and coronary artery disease. PPAR Res..

[43] Pfützner A., Schneider C.A., Forst T. (2006). Pioglitazone: An antidiabetic drug with cardiovascular therapeutic effects. Expert Rev. Cardiovasc. Ther..

[44] Pfützner A., Weber M.M., Forst T. (2007). Pioglitazone: Update on an oral antidiabetic drug with antiatherosclerotic effects. Expert Opin. Pharmacother..

[45] Betteridge D.J. (2009). CHICAGO, PERISCOPE and PROactive: CV risk modification in diabetes with pioglitazone. Fundam. Clin. Pharmacol..

[46] Pfützner A., Schöndorf T., Hanefeld M., Forst T. (2010). High-sensitivity C-reactive protein (hs-CRP) predicts cardiovascular risk in diabetic and non-diabetic patients: Effects of insulin-sensitizing treatment with pioglitazone. J. Diabetes Sci. Technol..

[47] Langenfeld M.R., Forst T., Hohberg C., Kann P., Lübben G., Konrad T., Füllert S.D., Sachara C., Pfützner A. (2005). Pioglitazone decreases carotid intima-media thickness independently of glycemic control in patients with type 2 diabetes mellitus: Results from a controlled randomized study. Circulation.

[48] Mazzone T., Meyer P.M., Feinstein S.B., Davidson M.H., Kondos G.T., D’Agostino R.B., Perez A., Provost J.C., Haffner S.M. (2006). Effect of pioglitazone compared with glimepiride on carotid intima-media thickness in type 2 diabetes: A randomized trial. JAMA.

[49] Polonsky T., Mazzone T., Davidson M. (2009). The clinical implications of the CHICAGO study for the management of cardiovascular risk in patients with type 2 diabetes mellitus. Trends Cardiovasc. Med..

[50] Murphy C.E., Rodgers P.T. (2007). Effects of thiazolidinediones on bone loss and fracture. Ann. Pharmacother..

[51] Kermani A., Garg A. (2003). Thiazolidinedione-associated congestive heart failure and pulmonary edema. Mayo Clin. Proc..

[52] Tang W.H., Francis G.S., Hoogwerf B.J., Young J.B. (2003). Fluid retention after initiation of thiazolidinedione therapy in diabetic patients with established chronic heart failure. J. Am. Coll. Cardiol..

[53] Chelliah A., Burge M.R. (2004). Insulin edema in the twenty-first century: Review of the existing literature. J. Investig. Med..

[54] Herrero M.H., Zaragoza N.R., Llop M.E., Rovira A.F., Mercader P.T. (2023). Insulin edema, a little known entity. Acta Diabetol..

[55] Custódio T.F., Paulsen P.A., Frain K.M., Pedersen B.P. (2021). Structural comparison of GLUT1 to GLUT3 reveal transport regulation mechanism in sugar porter family. Life Sci. Alliance.

[56] Kashani L., Omidvar T., Farazmand B., Modabbernia A., Ramzanzadeh F., Tehraninejad E.S., Ashrafi M., Tabrizi M., Akhondzadeh S. (2013). Does pioglitazone improve depression through insulin-sensitization? Results of a randomized double-blind metformin-controlled trial in patients with polycystic ovarian syndrome and comorbid depression. Psychoneuroendocrinology.

[57] Lin K.W., Wroolie T.E., Robakis T., Rasgon N.L. (2015). Adjuvant pioglitazone for unremitted depression: Clinical correlates of treatment response. Psychiatry Res..

[58] Colle R., de Larminat D., Rotenberg S., Hozer F., Hardy P., Verstuyft C., Fève B., Corruble E. (2017). PPAR-γ Agonists for the Treatment of Major Depression: A Review. Pharmacopsychiatry.

[59] Ikram H., Sheikh S.A., Haleem D.J., Ganau M., Choudhry A.M. (2021). Dose related acute behavioral and neurochemical profile of pioglitazone. Pak. J. Pharm. Sci..

[60] Zhu Z., Shen Z., Lu Y., Zhong S., Xu C. (2012). Increased risk of bladder cancer with pioglitazone therapy in patients with diabetes: A meta-analysis. Diabetes Res. Clin. Pract..

[61] Ferwana M., Firwana B., Hasan R., Al-Mallah M.H., Kim S., Montori V.M., Murad M.H. (2013). Pioglitazone and risk of bladder cancer: A meta-analysis of controlled studies. Diabetes Med..

[62] de Vries F., Zeegers M., Goossens M.E. (2013). Pioglitazone and bladder cancer: Two studies, same database, two answers. Br. J. Clin. Pharmacol..

[63] Ripamonti E., Azoulay L., Abrahamowicz M., Platt R.W., Suissa S. (2019). A systematic review of observational studies of the association between pioglitazone use and bladder cancer. Diabetes Med..

[64] Li Y.R., Liu C.H., Sun W.C., Fan P.Y., Liu F.H., Chen T.H., Wu V.C., Lin C., Hsiao C.C. (2021). The Risk of Bladder Cancer in Type 2 Diabetes Mellitus with Combination Therapy of SGLT-2 Inhibitors and Pioglitazone. J. Pers. Med..

[65] Ryder R.E.J., DeFronzo R.A. (2019). Pioglitazone: Inexpensive; very effective at reducing HbA1c; no evidence of bladder cancer risk; plenty of evidence of cardiovascular benefit. Diabetes Med..

[66] Stanley S., Wynne K., Bloom S. (2004). Gastrointestinal satiety signals III. Glucagon-like peptide 1, oxyntomodulin, peptide YY, and pancreatic polypeptide. Am. J. Physiol. Gastrointest. Liver Physiol..

[67] Wren A.M., Bloom S.R. (2007). Gut hormones and appetite control. Gastroenterology.

[68] Liu Q.K. (2024). Mechanisms of action and therapeutic applications of GLP-1 and dual GIP/GLP-1 receptor agonists. Front. Endocrinol..

[69] Nauck M.A., Quast D.R., Wefers J., Meier J.J. (2021). GLP-1 receptor agonists in the treatment of type 2 diabetes—State-of-the-art. Mol. Metab..

[70] Zheng Z., Li S., Liu M., Chen C., Cheng H., Deng H., Deng J., Li Y., Sun S., Huang J. (2024). Glucagon-like peptide-1 receptor: Mechanisms and advances in therapy. Signal Transduct. Target. Ther..

[71] Nauck M.A., Tornøe K., Rasmussen S., Treppendahl M.B., Marso S.P., LEADER Publication Committee on behalf of the LEADER Trial Investigators (2018). Cardiovascular outcomes in patients who experienced a myocardial infarction while treated with liraglutide versus placebo in the LEADER trial. Diabetes Vasc. Dis. Res..

[72] Vemulapalli H.S., Vajje J., Rehman W., Virk G.S., Shah K., Chaudhari S.S., Mian I.U., Saleem F. (2023). Safety and Efficacy of Liraglutide on Cardiovascular Outcomes in Patients With Diabetes Mellitus: A Meta-Analysis of Randomized Controlled Trials. Cureus.

[73] Aldawsari M., Almadani F.A., Almuhammadi N., Algabsani S., Alamro Y., Aldhwayan M. (2023). The Efficacy of GLP-1 Analogues on Appetite Parameters, Gastric Emptying, Food Preference and Taste Among Adults with Obesity: Systematic Review of Randomized Controlled Trials. Diabetes Metab. Syndr. Obes..

[74] Kabahizi A., Wallace B., Lieu L., Chau D., Dong Y., Hwang E.S., Williams K.W. (2022). Glucagon-like peptide-1 (GLP-1) signalling in the brain: From neural circuits and metabolism to therapeutics. Br. J. Pharmacol..

[75] Krieger J.P. (2020). Intestinal glucagon-like peptide-1 effects on food intake: Physiological relevance and emerging mechanisms. Peptides.

[76] Hellström P.M., Geliebter A., Näslund E., Schmidt P.T., Yahav E.K., Hashim S.A., Yeomans M.R. (2004). Peripheral and central signals in the control of eating in normal, obese and binge-eating human subjects. Br. J. Nutr..

[77] Buse J.B., Nauck M., Forst T., Sheu W.H., Shenouda S.K., Heilmann C.R., Hoogwerf B.J., Gao A., Boardman M.K., Fineman M. (2013). Exenatide once weekly versus liraglutide once daily in patients with type 2 diabetes (DURATION-6): A randomised, open-label study. Lancet.

[78] Crane J., McGowan B. (2016). The GLP-1 agonist, liraglutide, as a pharmacotherapy for obesity. Ther. Adv. Chronic Dis..

[79] Abdul-Ghani M.A., DeFronzo R.A. (2008). Inhibition of renal glucose reabsorption: A novel strategy for achieving glucose control in type 2 diabetes mellitus. Endocr. Pract..

[80] Chen L.H., Leung P.S. (2013). Inhibition of the sodium glucose co-transporter-2: Its beneficial action and potential combination therapy for type 2 diabetes mellitus. Diabetes Obes. Metab..

[81] March C.A., Libman I.M., Becker D.J., Levitsky L.L. (2022). From Antiquity to Modern Times: A History of Diabetes Mellitus and Its Treatments. Horm. Res. Paediatr..

[82] Barnett A.H. (2013). Impact of sodium glucose cotransporter 2 inhibitors on weight in patients with type 2 diabetes mellitus. Postgrad. Med..

[83] Ye Z.W., Wu X.M., Jiang J.G. (2009). Expression changes of angiotensin II pathways and bioactive mediators during human preadipocytes-visceral differentiation. Metabolism.

[84] Tikkanen I., Narko K., Zeller C., Green A., Salsali A., Broedl U.C., Woerle H.J., EMPA-REG BP Investigators (2015). Empagliflozin reduces blood pressure in patients with type 2 diabetes and hypertension. Diabetes Care.

[85] Pfeifer M., Townsend R.R., Davies M.J., Vijapurkar U., Ren J. (2017). Effects of canagliflozin, a sodium glucose co-transporter 2 inhibitor, on blood pressure and markers of arterial stiffness in patients with type 2 diabetes mellitus: A post hoc analysis. Cardiovasc. Diabetol..

[86] Alexander J.T., Staab E.M., Wan W., Franco M., Knitter A., Skandari M.R., Bolen S., Maruthur N.M., Huang E.S., Philipson L.H. (2022). Longer-term Benefits and Risks of Sodium-Glucose Cotransporter-2 Inhibitors in Type 2 Diabetes: A Systematic Review and Meta-analysis. J. Gen. Intern. Med..

[87] Wang C., Zhou Y., Kong Z., Wang X., Lv W., Geng Z., Wang Y. (2019). The renoprotective effects of sodium-glucose cotransporter 2 inhibitors versus placebo in patients with type 2 diabetes with or without prevalent kidney disease: A systematic review and meta-analysis. Diabetes Obes. Metab..

[88] Heidenreich P. (2024). Heart failure management guidelines: New recommendations and implementation. J. Cardiol..

[89] Pfützner A., Klonoff D., Heinemann L., Ejskjaer N., Pickup J. (2017). Euglycemic ketosis in patients with type 2 diabetes on SGLT2-inhibitor therapy-an emerging problem and solutions offered by diabetes technology. Endocrine.

[90] Forst T., Hohberg C., Pfützner A. (2009). Cardiovascular effects of disturbed insulin metabolism in metabolic syndrome and type 2 diabetes patients. Horm. Metab. Res..

[91] Kramer C.K., Zinman B., Retnakaran R. (2013). Short-term intensive insulin therapy in type 2 diabetes mellitus: A systematic review and meta-analysis. Lancet Diabetes Endocrinol..

[92] Li Y., Xu W., Liao Z., Yao B., Chen X., Huang Z., Hu G., Weng J. (2004). Induction of long-term glycemic control in newly diagnosed type 2 diabetic patients is associated with improvement of beta-cell function. Diabetes Care.

[93] Davies M.J., Aroda V.R., Collins B.S., Gabbay R.A., Green J., Maruthur N.M., Rosas S.E., Del Prato S., Mathieu C., Mingrone G. (2022). Management of hyperglycemia in type 2 diabetes, 2022. A consensus report by the American Diabetes Association (ADA) and the European Association for the Study of Diabetes (EASD). Diabetes Care.

[94] Tsur A., Harman-Bohem I., Buchs A.E., Raz I., Wainstein J. (2006). The guidelines for the diagnosis prevention and treatment of type 2 diabetes mellitus–2005. Harefuah.

[95] International Diabetes Federation Diabetes Atlas 2023. https://diabetesatlas.org/.

[96] Hellman B. (2009). Pulsatility of insulin release—A clinically important phenomenon. Upsala J. Med. Sci..

[97] Wahren J., Kallas Å (2012). Loss of pulsatile insulin secretion: A factor in the pathogenesis of type 2 diabetes?. Diabetes.

[98] Dailey G.E., Boden G.H., Creech R.H., Johnson D.G., Gleason R.E., Kennedy F.P. (2000). Effects of pulsatile intravenous insulin therapy on the progression of diabetic nephropathy. Metabolism.

[99] Weinrauch L.A., Sun J., Gleason R.E., Boden G.H., Creech R.H., Dailey G., Kennedy F.P., Weir M.R., D’Elia J.A. (2010). Pulsatile intermittent intravenous insulin therapy for attenuation of retinopathy and nephropathy in type 1 diabetes mellitus. Metabolism.

[100] Weinrauch L.A., Burger A.J., Aepfelbacher F., Lee A.T., Gleason R.E., D’Elia J.A. (2007). A pilot study to test the effect of pulsatile insulin infusion on cardiovascular mechanisms that might contribute to attenuation of renal compromise in type 1 diabetes mellitus patients with proteinuria. Metabolism.

[101] Manessis A., Hanna M.R., Sachsenheimer D., Do L., Lewin J.C., Steiner S.S., McCormack S., Demircik F., Pfützner A. (2021). Pulsatile Insulin Infusion as a Treatment Option for Patients with Type 2 Diabetes and Stage III Kidney Failure—Results from a Pilot Study. EC Endocrinol. Metab. Res..

[102] Feinman R.D., Pogozelski W.K., Astrup A., Bernstein R.K., Fine E.J., Westman E.C., Accurso A., Frassetto L., Gower B.A., McFarlane S.I. (2015). Dietary carbohydrate restriction as the first approach in diabetes management. Nutrition.

[103] Saslow L.R., Daubenmier J.J., Moskowitz J.T., Kim S., Murphy E.J., Phinney S.D., Ploutz-Snyder R., Goldman V., Cox R.M., Mason A.E. (2017). Twelve-month outcomes of a randomized trial of a moderate-carbohydrate versus very low-carbohydrate diet in overweight adults with type 2 diabetes. J. Nutr..

[104] Tay J., Luscombe-Marsh N.D., Thompson C.H., Noakes M., Buckley J.D., Wittert G.A., Yancy W.S., Brinkworth G.D. (2015). Comparison of low- and high-carbohydrate diets for type 2 diabetes management: A randomized trial. Am. J. Clin. Nutr..

[105] Meng Y., Bai H., Wang S., Li Z., Wang Q., Chen L. (2017). Efficacy of low carbohydrate diet for type 2 diabetes mellitus management: A systematic review and meta-analysis of randomized controlled trials. Diabetes Res. Clin. Pract..

[106] Hallberg S.J., McKenzie A.L., Williams P.T., Bhanpuri N.H., Peters A.L., Campbell W.W., Hazbun T.L., Volk B.M., McCarter J.P., Phinney S.D. (2018). Effectiveness and safety of a novel care model for the management of type 2 diabetes at one year: An open-label, non-randomized, controlled study. Diabetes Ther..

[107] Colberg S.R., Sigal R.J., Yardley J.E., Riddell M.C., Dunstan D.W., Dempsey P.C., Horton E.S., Castorino K., Tate D.F. (2016). Physical Activity/Exercise and Diabetes: A Position Statement of the American Diabetes Association. Diabetes Care.

[108] Wan Y., Li J., Sun S., Li X., Wang R., Liu X., Zhang F., Zhao Y. (2024). The impact of resistance exercise training on glycemic control among adults with type 2 diabetes: A systematic review and meta-analysis of randomized controlled trials. J. Diabetes Sci. Technol..

[109] Ahlqvist E., Storm P., Käräjämäki A., Martinell M., Dorkhan M., Carlsson A., Vikman P., Prasad R.B., Aly D.M., Almgren P. (2018). Novel subgroups of adult-onset diabetes and their association with outcomes: A data-driven cluster analysis of six variables. Lancet Diabetes Endocrinol..

[110] Chung W.K., Erion K., Florez J.C., Hattersley A.T., Hivert M.F., Lee C.G., McCarthy M.I., Nolan J.J., Norris J.M., Pearson E.R. (2020). Precision Medicine in Diabetes: A Consensus Report from the American Diabetes Association (ADA) and the European Association for the Study of Diabetes (EASD). Diabetes Care.

[111] Misra S., Wagner R., Ozkan B., Schön M., Sevilla-Gonzalez M., Prystupa K., Wang C.C., Kreienkamp R.J., Cromer S.J., Rooney M.R. (2023). Precision subclassification of type 2 diabetes: A systematic review. Commun. Med..

[112] Pfützner A., Jantz J., Rose D.M. (2025). Successful Personalized Type 2 Diabetes Management for Airline Pilots by Means of ß-Cell-protective De-Escalation Treatment (DET)—Background and Case Reports. Arch. Clin. Case Stud..

